# Early career researchers want Open Science

**DOI:** 10.1186/s13059-017-1351-7

**Published:** 2017-11-15

**Authors:** Andrea Farnham, Christoph Kurz, Mehmet Ali Öztürk, Monica Solbiati, Oona Myllyntaus, Jordy Meekes, Tra My Pham, Clara Paz, Magda Langiewicz, Sophie Andrews, Liisa Kanninen, Chantal Agbemabiese, Arzu Tugce Guler, Jeffrey Durieux, Sarah Jasim, Olivia Viessmann, Stefano Frattini, Danagul Yembergenova, Carla Marin Benito, Marion Porte, Anaïs Grangeray-Vilmint, Rafael Prieto Curiel, Carin Rehncrona, Tareq Malas, Flavia Esposito, Kristina Hettne

**Affiliations:** 1 Epidemiology, Biostatistics and Prevention Institute, University of Zurich, 8001 Zurich, Switzerland; 2 Institute of Health Economics and Health Care Management, Helmholtz Zentrum München, 85764 Oberschleissheim, Neuherberg Germany; 3 Institute for Medical Information Processing, Biometry and Epidemiology (IBE), Ludwig-Maximilians-Universität München, 81377 Munich, Germany; 4 Heidelberg Institute for Theoretical Studies, 69118 Heidelberg, Germany; 5 Fondazione IRCCS Ca’ Granda, Ospedale Maggiore Policlinico, Università degli Studi di Milano, 20122 Milan, Italy; 6 University of Helsinki, 00100 Helsinki, Finland; 7 Utrecht University School of Economics, Kriekenpitplein 21-22, 3584 EC Utrecht, The Netherlands; 8 University College London, London, NW3 2PF UK; 9 Universitat de Barcelona, 08007 Barcelona, Spain; 10 University Hospital Zurich, Faculty of Medicine, 8091 Zurich, Switzerland; 11 University of Oxford, Oxford, OX1 2JD UK; 12 Nagasaki University, Graduate School of Biomedical Sciences, Nagasaki, 852-8523 Japan; 13 Center for Proteomics and Metabolomics, Leiden University Medical Center, 2333 ZA Leiden, The Netherlands; 14 Leiden University, Faculty of Social and Behavioural Sciences, 2333 AK Leiden, The Netherlands; 15 Faculty of Medicine, Imperial College London, London, SW7 2AZ UK; 16 University of Milan, 20133 Milan, Italy; 17 University of Geneva, Faculty of Education, 1205 Geneva, Switzerland; 18 Université Pierre et Marie Curie, 75005 Paris, France; 19 University of Strasbourg, 67081 Strasbourg, France; 20 Lund University, Campus Helsingborg, 252 08 Helsingborg, Sweden; 21 Leiden University Medical Center, 2333 ZA Leiden, The Netherlands

## Abstract

Open Science is encouraged by the European Union and many other political and scientific institutions. However, scientific practice is proving slow to change. We propose, as early career researchers, that it is our task to change scientific research into open scientific research and commit to Open Science principles.

## The Open Science situation

For 300 years, research journals have provided a stable record of the main conclusions of scientific studies, the methods, and the contact details of the scientists from whom data and materials might be obtained upon request. As the volume and complexity of research data explode, traditional research publications are failing to pay even lip service to the task of indexing data, let alone contribute meaningfully to data stewardship and preservation. Despite widespread discussion of the importance of Open Science and a growing recognition of the need for more sophisticated data stewardship practices, such as those in the FAIR (Findable, Accessible, Interoperable and Reusable) guidelines [1], the research community continues to do little to use available tools to index or share our vast datasets. The consequence is an escalation of data loss in an era when “data is the new gold” [2]. Although some in the scientific community see Open Science and data sharing as a “threat”, even labeling those who use others’ data as parasites [3], we believe that embracing and improving the Open Science tools already in place will facilitate better science, allowing us to harness the full potential of growing global scientific output.

Early career scientists, as relative outsiders to the scientific establishment, are often seen as dispensable, low-risk “experiments” but, working together to create interoperable systems, we have the opportunity to create change. Early career researchers have the least commitment toward professional hierarchy and are highly involved in data collection and analysis. Training young researchers to use the tools of Open Science can instigate a more reliable change in data stewardship. Here, we want to emphasize the importance of the implementation of Open Science principles across disciplines from a young researcher’s point of view, and highlight the reasons why young researchers are the key for change.

As a group of early-career researchers who convened for the 2016 LERU Doctoral Summer School on Data Stewardship, we commit to: (1) the growth of an Open Science framework within which we can explicitly receive credit for and give credit to datasets with machine-readable metadata, provenance, and reproducible workflows; (2) establish training in the principles of Open Science and the relevant software and communication tools; (3) a pledge to be the first generation that will pass on the principles and mindset of Open Science to the next generation. If these three aims become a reality, we also envisage a strengthening of the credibility and reproducibility of scientific findings, and a reduction in fraudulent scientific data.

## Growth of an Open Science framework

What does an Open Science framework look like? The European Commission on Open Science outlines a high-level vision of a future of science that includes Open Access, collaboration on platforms such as the European Open Science cloud, and the development of alternative metrics for measuring the impact of good science [4]. However, the roadmap to this future remains unclear to the everyday scientist working within the traditional scientific framework, and these excellent guidelines remain largely theoretical for many. The key to the growth of this Open Science framework is the cultivation of good data stewardship practices at every level of science, which conveniently is an actionable step for almost every scientist.

Data stewardship, a concept that involves all those data management issues related to long-term data reusability and interoperability, requires careful planning and thought from the beginning of a research project. Publication of data and code alongside traditional research papers, while widely done, is only the first step toward “FAIRifying” research. Perhaps more important is the creation of metadata on top of these datasets and code. It is from such a database of metadata that we will begin to drive innovation by identifying which datasets intersect well to produce results, and to create an executable data-code environment that can be peer-reviewed, built on, and reused. By changing research practices to include the creation and publication of such metadata, scientific culture will begin to change organically from the traditional focus on the static unit of the research paper to more dynamic, collaboration-based science. The growth of such a network of metadata will also provide the basic framework for the development of alternative metrics, such as precise citation to code versions, datasets, models, and the ability to quantify specific opinions and theories via the Semantic Web.

Despite its importance, data stewardship is often neglected until the end of a research project, when funding and time may be running low. The stewardship of data should be managed from the beginning of the study and included in the financial plan, as recognized by the EU recommendation for a 5% budget allocation for all funded research projects [5]. Producing research that complies with FAIR principles is an ethical responsibility for all scientists, and a plan for reuse should be an obligatory and fundamental part of study design, especially for those working with public funding. Beyond the ethical responsibility to produce transparent and reproducible research, young scientists today should view cultivation of data stewardship skills as an opportunity to participate in the exciting, innovative research of tomorrow.

## Overcoming the barriers to Open Science: a road map for young researchers

### Establishment of training in Open Science

The creation of truly FAIR research requires high-level understanding of the fundamental tenets and tools of Open Science. The ideal time to build these skillsets is early in the research career, when the structured training of young researchers offers the ideal opportunity to incorporate standardized training on skills in data stewardship into existing curricula. Resources such as the European-funded project *Facilitate Open Science Training for European Research* (FOSTER) [6] already provide online courses in four key areas of Open Science: open access, open data, open source, and open reproducible research. Short workshops in open access publishing options and modern scientific computing practices will promote open thinking within institutes about how to develop and improve their use of key Open Science tools.

### Avoid reinventing the wheel

The first and most fundamental step for researchers looking to change the way research is done in their field is to investigate what has already been done by their colleagues. What are the sharing platforms, available software, and standards? Is there an already existing ontology that can be referenced, making your data findable and interoperable? Avoid creating terminology when it already exists. Similarly, are there existing datasets and analytical pipelines that can help toward answering your research question? Working within the existing scientific framework can provide an opportunity to foster collaboration and avoid duplication of research effort.

Training in publishing more and better research papers, data-code objects, patents, and citable models based on open science principles should be standardized across fields and could be administered through existing infrastructures, such as Elixir, the European life science data organization. Scientific tools and sharing platforms that have emerged in recent years are Dataverse [7], Figshare [8], Dryad [9], Mendeley data [10], Zenodo [11], DataHub [12], DANS [13] and EUdat [14], Open Science Framework [15], and GitHub [16].

### Small steps move you forwards

Practicing FAIR data principles is not a binary state, but instead a matter of setting and achieving desirable standards for data sharing. The culture of data sharing begins within your own research team. A good first step for creating an Open Science environment in your workplace is to survey your own team on their own views and hesitations about data sharing, as well as establishing where it lies on their list of priorities. This can open an important dialogue and help identify concrete actions that your team can take to begin to move toward Open Science.

To facilitate the transition, we provide a summary box with practical advice for young researchers to engage in Open Science that require very little effort (Box 1).

## Box 1. How to engage in Open Science today

1. Submit pre-prints of your manuscript to publicly available repositories. Many major journals allow the posting of pre-prints to open repositories, e.g. arxiv.org, prior to submission and peer review.

2. Post published articles in a public repository (e.g. Pubmed Central). Typically, 6–12 months after publication, most publishers allow the posting of an author’s version of the manuscript to public repositories.

3. Publish in open access journals where possible. Many subscription-based journals also offer the option to pay an additional charge for open access.

4. Share data and material. The code, methods, and data to produce findings in your manuscript should be made publicly available in an open repository equipped with credit metrics for data generator, code writer, and data reuser. These metrics should be based upon real and precise utility and should be transparent so that others can derive their own metrics from them.

## A pledge to be the first generation to pass on Open Science to our succeeding generation

Publishing openly is associated with higher citation rates [17]. Sharing data is becoming mandatory for increasing numbers of high-profile journals and funders [18, 19], and offers a citation advantage [20]. Open practices make it easier to connect with other researchers, facilitating visibility and access to novel data and software resources, and creating opportunities to communicate and contribute to ongoing communal projects [21].

Open access policies are championed across the European Research Area, and prominently feature in the recommendations of Horizon 2020, the European Commission’s research and innovation program [22]. Yet, from an early career researcher perspective, we do not see much change, and are often trapped in the hamster wheel of bad practices and habits endorsed by supervisors and colleagues. The onus is therefore on us to establish principles of Open Science and good data stewardship, and pass this on to succeeding generations. By taking a stand early in our research careers and choosing to seek ways to make our research FAIR from the very beginning, we have the power to effect a change in scientific culture from the ground up, making Open Science a reality instead of an ideal.
